# Evaluating the Feasibility of Hydrogel-Based Neural Cell Sprays

**DOI:** 10.3390/jfb14100527

**Published:** 2023-10-19

**Authors:** Daisy Evans, Aina Mogas Barcons, Raja Haseeb Basit, Christopher Adams, Divya Maitreyi Chari

**Affiliations:** 1 Keele University School of Medicine, Keele University, Staffordshire ST5 5BG, UK; daisy.evans2@uhnm.nhs.uk; 2 Department of Physiology, Anatomy and Genetics, Oxford Parkinson’s Disease Centre, University of Oxford, Oxford OX1 3AZ, UK; aina.mogasbarcons@dpag.ox.ac.uk; 3 Department of General Surgery, Queen Elizabeth Hospital, Birmingham B15 2GW, UK; raja.basit@nhs.net; 4 Neural Tissue Engineering, School of Life Sciences, Keele University, Staffordshire ST5 5BG, UK; c.adams@keele.ac.uk

**Keywords:** collagen hydrogel, transplant cell, spray delivery, cell delivery, neural transplantation

## Abstract

Neurological injuries have poor prognoses with serious clinical sequelae. Stem cell transplantation enhances neural repair but is hampered by low graft survival (<ca. 5%), necessitating the development of approaches to enhance post-transplant cell viability. Intracerebral injection exerts high mechanical forces on transplant cells with risks of haemorrhage/infection. Transplant cell sprays can offer a non-invasive alternative. This study has assessed if the addition of protective, encapsulating polymer hydrogels to a cell spray format is feasible. Hydrogels (0.1% (1 mg/mL), 0.3% and 0.6% type I rat tail collagen) were trialled for spray deliverability. Cell-enriched hydrogels (containing mouse cortical astrocytes) were sprayed onto culture substrates. Astrocyte viability, cell-specific marker expression, morphology and proliferation were assessed at 24 h and 72 h post spraying. Intra-gel astrocytes and hydrogels could be co-stained using a double immunocytological technique (picrosirius red (PR)/DAB-peroxidase co-labelling). Astrocyte viability remained high post spraying with hydrogel encapsulation (>ca. 80%) and marker expression/proliferative potential of hydrogel-sprayed astrocytes was retained. Combining a cell spray format with polymer encapsulation technologies could form the basis of a non-invasive graft delivery method, offering potential advantages over current cell delivery approaches.

## 1. Introduction

The complexity of neurological injuries with the intrinsic lack of central nervous system (CNS) regeneration means the clinical prognosis is often poor [1], with physical, cognitive, and emotional deficits [2]. Exogenous stem cell transplantation can enhance neural regeneration in several pathologies including spinal cord injury (SCI), ischaemic/haemorrhagic strokes, neurodegenerative diseases and traumatic brain injury (TBI) [3,4,5,6,7]. Despite demonstrable preclinical benefits, graft cell survival remains poor (<ca. 5%) [8,9]. Cell death following host rejection is a major contributing factor, but the method of cell delivery may itself influence transplant cell survival [9,10].

Safe and effective cell delivery to neurological injury sites is still a major clinical challenge. Cell tracking studies show that only 1.5–3.7% of intravenously infused mesenchymal stem cells (MSCs) reach the arterial system and 0.0005% reach cerebral tissue [11,12]. Direct cell injection into the CNS demonstrates poor long-term survival of graft cells. The injection of neural stem cells (NSCs) in mouse models of brain ischaemia resulted in the survival of only ~2% of grafted cells 28 days post transplantation and 1.1% of NSCs differentiating into NeuN-positive neurons (0.2 μL; 5 × 104 cells/μL) [13]. Similarly, only 1–3% of injected NSCs survived transplantation in a rat model of ischaemia (5 × 104 cells/μL in 1.5 μL suspension, after hippocampal injection) [14].

Injection parameters and shear forces exerted upon cells during the injection process could be a major contributor to poor cell survival [15]. Smaller bores of needles limit the passage of cells with cell clumping, mechanical deformation against needle walls, and reduced post-injection viabilities observed [15,16]. The physical blockage of needle cannulae has been observed during injection in clinical trials of cell transplantation [17,18,19]. In instances of clogging, greater levels of force are required to dislodge cell masses which may exacerbate shear forces on cells. Mechanical deformation may be further exacerbated following cell injection into densely packed neural tissue. Discrete bolus injections may contribute to the poor homogeneity of delivered cells, whilst invasive needle penetration may exacerbate neuroinflammation around the site of implantation, with further risks of haemorrhage and infection [4]. Such limitations with injection methods highlight the need for alternative cell delivery approaches to improve safety and clinical efficacy. Cell sprays offer a means of circumventing these limitations. They have the advantage of reduced traumatic cell deformation versus narrow-bored needles, non-invasive and homogenous cell delivery over wide and non-uniform tissue areas and the option for sprayed cell layering to enhance delivered cell numbers [17,20]. Being a minimally invasive approach, the risk of infection and haemorrhage is low and predictable clinical complications are minimal [17].

Woods et al. (2021) demonstrated the feasibility of a spray approach for the delivery of neural transplant cells [20]. Whilst neural cell spray feasibility was demonstrated in this study, a reduction in the viability of sprayed cell populations versus controls was evident [20]. Graft cell viability correlates directly with neurological outcomes, so the optimisation of cell spray delivery is required for improved clinical translation. 

Hydrogels can be utilised to enhance the efficacy of stem cell transplantation therapies [21]. The addition of an encapsulating biopolymer into cell sprays may offer a solution to achieve enhanced protection for transplanted cell populations. It is well established that hydrogel encapsulated delivery of transplant populations improves cell survival. For example, hyaluronan–heparin–collagen hydrogels as protective carriers significantly improve the survival of neural progenitor cells when injected into stroke infarcts in mouse models [22]. 

The current low viability of transplant cell populations in clinical trials is owed in part to transplant cell death secondary to host immune responses [23]. Transplant cell isolation from inflammatory reactions in a hydrogel (which mimics physiological conditions required for neuronal growth) may reduce inflammation-mediated graft cell death [24]. Their elasticity, high water content and biodegradability mean hydrogels demonstrate good biocompatibility, mimicking native healthy CNS tissues and supporting the exchange of nutrients and metabolites [24,25,26]. This could be particularly beneficial in penetrating traumatic brain injuries where hydrogels could act as an artificial extracellular matrix to promote cellular ingrowth into necrotic cavities [27]. Their ability to be modified can further enhance the efficacy of cell transplantation. For example, collagen hydrogels functionalised with vascular endothelial growth factor mimetic peptide KLT inhibited glial scar formation and enhanced angiogenesis when implanted into mice TBI cavities [28].

Hydrogels may also offer mechanical protection during the spray process by acting as a compressible barrier, meaning the direct transmission of shear forces onto cell membranes can be reduced. For example, Aguado et al. using the injection of human umbilical vein endothelial cells reported a significantly higher viability of cells when injected with crosslinked alginate hydrogels versus buffer alone (88.9% ± 5.0% versus 58.7% ± 8.1%) [29]. Hydrogels have also been shown to act as carriers for antibiotics, which could be utilised to reduce the incidence of infection in penetrative TBI [30]. 

Several materials have been utilised to form biocompatible hydrogels for cell transplantation. As the most abundant collagen protein in mammals, type 1 collagen demonstrates similarity with extracellular matrix and high biocompatibility [31,32]. It has been detected within the subventricular zone of the CNS and can support neural cell attachment and axonal growth as it possesses cell adhesion and signalling domains [33]. Collagen formulations are also used in existing neurosurgical procedures including sealants used for dural repair, suggesting compatibility with current devices [34]. Previous studies have demonstrated the feasibility of encapsulating neural transplant cells within type 1 collagen biomatrices [35], making this an ideal polymer to test cell encapsulation applications.

Combining the mechanobiological protection of a hydrogel biopolymer with the benefits of neural spray systems may enhance the efficacy of neural cell transplantation but this is yet to be explored. We hypothesise this approach could offer a method of augmented cell delivery. The aim of this study was to assess the feasibility and safety of developing a hydrogel (collagen) biomatrix-based neural cell spray. Our objectives were to: (a) identify a biopolymer concentration that retains the capacity for aerosolisation and gelation; (b) assess the ability to spray and encapsulate neural cells within a biomatrix; and (c) evaluate the viability and physiological normalcy of sprayed (encapsulated) neural cell populations. To facilitate the analyses, a double histochemical protocol combining immunocytochemistry with dye staining was developed and tested, to co-visualise encapsulated cells and the hydrogel biomatrix.

## 2. Materials and Methods

### 2.1. Materials

Cell culture reagents and cell culture grade plastics were from Thermo Fisher Scientific^TM^ (TFS) (Loughborough, UK) or Sigma-Aldrich^TM^ (Dorset, UK) unless otherwise stated. The following were used: DMEM (Dulbecco’s Modified Eagle Medium) (TFS^TM^, 41966), Fetal Bovine Serum (Gibco, TFS^TM^, 11573397), Penicillin-Streptomycin (Gibco, TFS^TM^, 15070063), Calcein AM (Calcein acetoxymethyl ester) (Corning^TM^, Tewkesbury, MA, USA), Ethidium Homodimer-1 (Sigma-Aldrich^TM^, E1903), Click-iT^®^ EdU Cell Proliferation Kit, Alexa Fluor^TM^ 594 dye (Invitrogen^TM^, TFS^TM^, C10339), Normal Goat Serum (NGS) (Jackson ImmunoResearch^TM^, Cambridge, UK, 005000001), Normal Donkey Serum (NDS) (Jackson ImmunoResearch^TM^, Cambridge, UK, 170000001), Collagen (type 1 rat tail, Corning^®^, Tewskesbury, MA, USA, 354236), Glutamax-1 Supplement (Gibco^TM^, TFS^TM^, 35050038), Sodium pyruvate (Sigma-Aldrich^TM^ S8636), TryplE Express Enzyme (TFS^TM^, 12605028), Poly-D-Lysine (Sigma-Aldrich^TM^ P6407), VECTASTAIN^®^ ABC Reagent (Peroxidase) (Vector Laboratories, Newark, CA, USA, PK-6100), Picrosirius Red (Abcam^TM^, Cambridge, UK, ab246832), Glial fibrillary acidic protein (GFAP) rabbit antibody (Agilent, Santa Clara, CA, USA, Z0334), Donkey anti-rabbit (FITC) secondary antibody (Jackson Immuno Research^TM^, Cambridge, UK, 711095152), mounting medium with 4′,6-diamidino-2-phenylindole (DAPI) from Vectashield® (Vector Laboratories, Newark, CA, USA, H-1000) and Minimal Essential Medium (MEMx) (TFS^TM^, 12492013). Mist plastic pump spray bottles (10 mL) were utilised as spray devices and sourced from SelfTek^TM^ (Guangdong, China).

### 2.2. Hydrogel Spray Preparation

**Figure 1:**
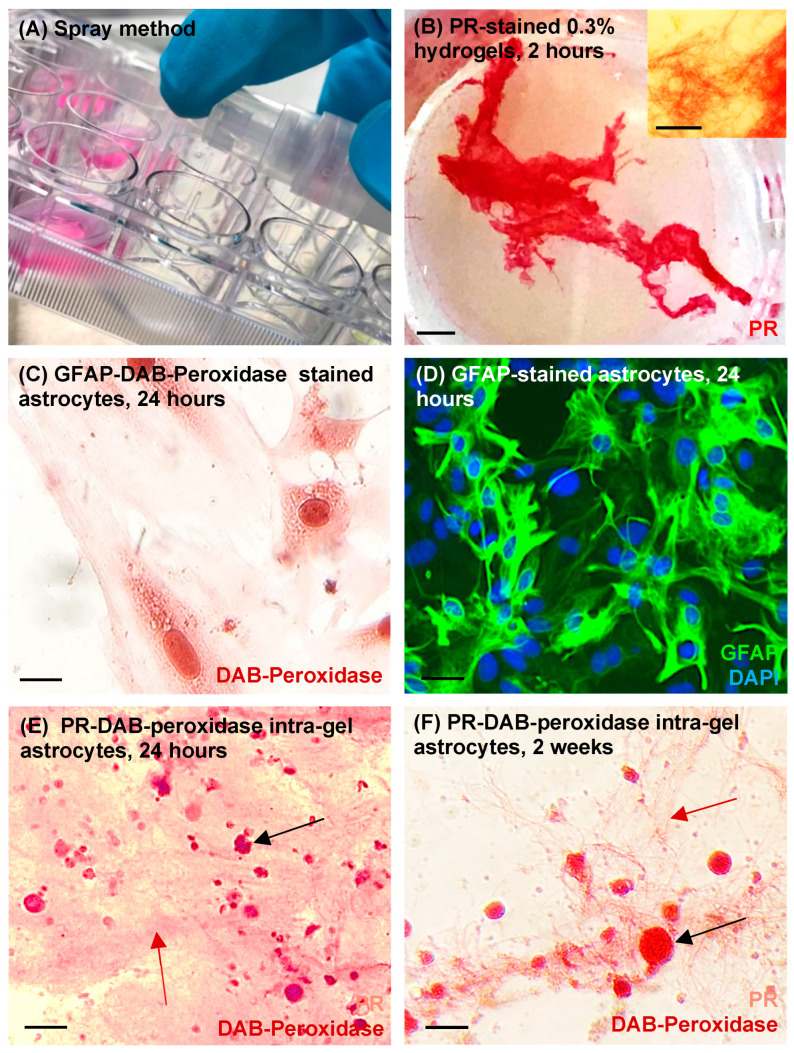
**Technical validation of methods and staining techniques.** (**A**) Hydrogels of varying collagen concentrations were trialled and sprayed onto culture well surfaces to qualitatively assess optimum collagen concentration. (**B**) 0.3% hydrogels sprayed into culture wells showed observable gelation properties after incubation for 2 h at 37 °C and staining with picrosirius red (PR), scale bar 2 mm, forming a fibrillary matrix mesh visible with light microscopy (inset), scale bar 50 μm. (**C**) GFAP-DAB-peroxidase staining was technically validated on sprayed (minus hydrogel) glass-adhered astrocytes which showed characteristic flattened, polygonal astrocytic morphologies, scale bar 5 μm. (**D**) GFAP staining was technically validated on sprayed (minus hydrogel) glass-adhered astrocytes (unstained cells may represent other glial cell types that were not removed during the mixed glial culture process), scale bar 25 μm. (**E**,**F**) Representative image of technical validation of PR-DAB-peroxidase co-staining method where (**E**) shows immunocytochemical co-staining of intra-gel hydrogel sprayed astrocytes at 24 h post spray: PR-stained hydrogel fibres (red arrow) and DAB-stained spherical astrocyte clusters (black arrow), scale bar 50 μm. (**F**) immunocytochemical co-staining of intra-gel hydrogel sprayed astrocytes at 2 weeks post spray, PR-stained hydrogel fibres (red arrow) and DAB-stained spherical astrocyte clusters (black arrow), scale bar 50 μm.

In preliminary trial studies, hydrogel solutions of varying collagen concentrations (type 1 rat tail collagen, Corning™) were qualitatively trialled to determine the optimum concentrations to yield a gel that could retain both aerosolisation and gelation capacities. Collagen hydrogel solutions of 0.1% (*w*/*v*%), 0.3% (*w*/*v*%) and 0.6% (*w*/*v*%) were prepared with varying reagents (MEMx (10×), collagen (3–4 mg/mL), acetic acid (0.023 M), NaOH (1 M)) and sprayed onto culture well surfaces (Figure 1A). Various parameters were qualitatively assessed (Table 1). These were: spray resistance, spray volume consistency, tubing blockage and gelation capacity at 37 °C after spraying. This was performed to establish which hydrogels offered minimal spray resistance, and achieved even spray consistency with minimal blockage of the tubing with the spray device utilised.

### 2.3. Derivation of Primary Astrocyte Cultures

Mixed glial cultures (MGCs) were obtained from P1-3 mice cortices. All experiments were approved by the local ethical committee. Mice were humanely euthanised under pentobarbital anaesthesia overdose (schedule 1 procedure), with brains transferred to dissection medium (Hank’s balanced salt solution, HEPES, 50 U/mL penicillin, 50 μg/mL streptomycin) on ice. Olfactory bulbs and hindbrains were removed, and minced cortices suspended in D10 medium (43.5 mL Dulbecco’s Modified Eagle Medium (DMEM), 0.5 mL Glutamax-1 1%, 0.5 mL Penicillin–Streptomycin, 5 mL sodium pyruvate, 5 mL 10% BSA). Following trituration, cells were strained (70 μm strainer) and suspended in D10 (density 2 × 10^5^ cells/mL). Cells were then seeded into poly-D-Lysine (PDL)-coated T175 flasks and incubated at 37 °C until confluent MGCs were achieved at day seven.

At day 10, flasks were placed onto a rotary shaker (37 °C) at 220 rpm (2 h) to detach microglia. Flasks were then re-gassed in incubators (37 °C) for six hours and placed onto the rotary shaker overnight (220 rpm) to remove oligodendrocyte precursor cells (OPCs) only. Only primary astrocytes subsequently remained within the flasks. To remove attached astrocytes, 15–20 mL of TryplE (1 X) was added to each flask and placed onto a rotary shaker (220 rpm, four minutes). Once cells started to detach, 5 mL D10 was immediately added to stop trypsinisation and the cell solution was collected. These primary astrocytes were cultured onto 24-well plates containing poly-D-Lysine-coated coverslips.

### 2.4. Cell Delivery

For cell delivery, the methods included spraying astrocytes mixed with 0.3% collagen solution and spraying astrocytes alone (plastic 10 mL pump spray device). The total volume of neural cell solution within the spray device was 4 mL at a density of 2.4 × 10^5^ cells/mL. For hydrogel sprays, this comprised of 2 mL of cell suspension (0.8 mL cell-free D10 medium plus 1.2 mL astrocyte suspension in D10) mixed with 2 mL hydrogel solution. Three sprays were delivered per well to give an estimated total volume of 360 μL cell solution per well. Astrocytes were delivered with a final cell density of 2.4 × 10^5^ cells/mL per well. Final cell density and total volume within each well were the same across both delivery methods, as cell density has been shown to influence cell viability. The possibility of cell density as a confounder was therefore avoided. Hydrogel cell solutions were sprayed at a height of 1.4 cm from the well floor (closest possible distance). Due to the aerosolisation of cell suspensions, a ‘checkerboard’ pattern of wells was utilised to prevent contamination of sprayed cells into neighbouring wells. Following plating, pipetted medium was used to pool any remaining cells sprayed onto the walls of each well.

All plates were cultured at 37 °C 5% CO_2_/95% humified air with a 50% change of D10 medium performed every 2–3 days. At each assay time point, astrocytes were fixed in 4% paraformaldehyde (PFA) for 1 h and washed with PBS.

### 2.5. Cell and Hydrogel Biomatrix Co-Visualisation Using Double Staining

To co-visualise intra-gel astrocytes and surrounding hydrogel biomatrices, we established a protocol of picrosirius red (PR)-3,3′-diaminobenzidine (DAB)-based peroxidase staining which was performed at 24 h and at 2 weeks (the latter to identify gel degradation). Post fixation with PFA, sprayed hydrogels containing astrocytes were washed in buffer (0.3% triton in phosphate-buffered saline (PBS)) and incubated in diluted 5% normal goat serum (NGS) (20 min). Hydrogels were then incubated with primary antibody solutions overnight in a blocking solution (5% NGS, 0.3% Triton-X in PBS) (glial fibrillary acidic protein (GFAP) rabbit, dilution factor 1:500 Ab:blocker). They were then washed in buffer and incubated for 30 min with diluted biotinylated secondary antibody solution (donkey anti-rabbit GFAP, dilution factor 1:200). After washing with buffer, VECTASTAIN^®^ ABC Reagent was added (30 min) and again washed with buffer. DAB-peroxidase substrate was then prepared (84 μL buffer stock solution, 100 μL DAB stock solution, 80 μL hydrogen peroxide solution, 80 μL nickel solution) and incubated with hydrogels until the desired intensity of staining developed (2–10 min). DAB solutions were washed off with tap water to avoid inhibition of peroxidase reactions which may occur with deionised water. Finally, collagen fibres were stained by incubating in 100% PR (30 min) before the excess was removed with tap water. Specimens were visualised under light microscopy using an axioscope Zeiss microscope.

### 2.6. Assessment of Astrocyte Viability 

Live–dead assays (Ethidium Homodimer-1 (EthD-1)/Calcein AM) were performed at 24 and 72 h to evaluate the survival of astrocytes post delivery. During live–dead assays, cell-permeable calcein AM becomes modified into a fluorescent form by intracellular esterase activity to identify living cells, whilst EthD-1 selectively stains cellular DNA of dead cells due to a loss of their membrane integrity [36]. This method has been determined to be a reliable method of cell viability assessment with comparable results to flow cytometry [36]. EthD-1 was therefore used to visualise dead astrocytes and calcein-AM was used to visualise live astrocytes. Live–dead reagents were diluted in DMEM cell culture media (3 μL/mL EthD-1, 1 μL/mL of calcein AM) and 300 μL of the resultant solution added to each well. Plates were incubated at 37 °C for 20 min before being washed with PBS three times. During each experimental method, the washing of cell cultures was performed to (a) remove excess reagents from each step and (b) due to the need for specific reagent reaction times. This was to prevent non-specific fluorescence staining and artefacts and to stop the reaction at the specified time. 

### 2.7. Assessment of Astrocyte Proliferation

To assess astrocyte proliferative capacity post delivery, Click-iT 5-Ethynyl-2′-deoxyuridine (EdU) assays were performed at 72 h. A total of 1 μL/mL EduA reagent was mixed with cell medium and added to each well (100% medium change). Plates were incubated at 37 °C for 6 h before being washed with PBS (3 washes) and fixed. Astrocytes were then washed in 0.3% Triton in PBS for 40 min then twice with 3% bovine serum albumin (BSA). EdU reagent solution was then prepared (EdU D 860 μL/mL, EdU E 40 μL/mL, EdU B 2.5 μL/mL, EdU F 100 μL/mL (dilution factor 1:10 in dH_2_O) and 300 μL of the solution was added to each well for 1 h in the dark at room temperature (RT). Astrocytes were subsequently washed twice in 3% BSA then PBS (3 washes).

### 2.8. Assessment of Astrocyte-Specific Protein Marker (GFAP) Expression

Immunocytochemistry was performed to identify astrocyte-specific marker expression (GFAP) at 72 h post spray. After fixing, blocking solution was added for 30 min (10% Normal Donkey Serum (NDS) in 0.3% Triton X-100 in PBS). Primary antibodies (GFAP rabbit) were diluted in blocking solution to a factor of 1:500 (Ab:blocker). These were added to samples and incubated overnight at 4 °C in the dark. Primary antibodies were removed, samples were washed three times in PBS, and blocking solution was added after 30 min. Secondary antibodies (donkey anti-rabbit (FITC)) were prepared at a dilution of 1:200 in blocker and added to samples for 2 h at RT in the dark. Primary antibodies were removed with three PBS washes. 4′6-diamidino-2-phenylindole (DAPI) solution was added to the second of the final washes with phosphate-buffered saline (PBS) (1 μL/mL) to stain all cell nuclei.

### 2.9. Cell imaging, Quantification and Statistical Analysis

Specimens were imaged with fluorescence microscopy within PBS-filled wells using a Zeiss Z-stack Axio Observer Z1 microscope equipped with AxioCam (Zen 2) and AxioVision software. Images were merged using Image J (version 1.53). Images were analysed using an Image-J cell counter tool. GraphPad Prism 9.0.0 software was utilized for all statistical analyses. To assess astrocyte viability and proliferation, 8 random fields at ×40 magnification were digitally captured. Total cell numbers per field (DAPI-stained nuclei) and total dead cells (EthD-1-stained cells) per field/proliferating (EdU-stained cells) per field were counted using Image-J software to calculate the proportion of dead cells and proliferating cells per field, respectively. Although cells showed a tendency to form glial cell clusters, the ease of identification of nuclei within each cluster made it possible to reliably obtain cell counts. For each experiment, a total of three biological repeats were performed (*n* = 3) with cells derived from different mouse litters. Averages were calculated for each delivery method at each time point and charts were generated using GraphPad software representing means ± standard deviation with error bars. Statistically significant differences between groups were calculated (* = 0.01 > *p* < 0.05, ** = 0.001 > *p* < 0.01, *** = 0.0001> *p* < 0.001). Data were analysed for statistically significant differences using non-parametric Mann–Whitney tests.

## 3. Results

### 3.1. 0.3% Collagen Solutions Showed Optimal Properties for Spray Delivery: Co-Staining Revealed Cell Clusters within the Biomatrix

In qualitative trial experiments using light microscopy, it was evident that 0.1% collagen solutions possessed minimal observable gelation properties post spray and demonstrated a liquid consistency (Table 1). By contrast, 0.6% collagen demonstrated spray resistance, blockage of tubing and inconsistent, low-volume sprays. 0.3% collagen solution demonstrated minimal spray resistance and retained gelation properties. Visualisation was enhanced with the use of picrosirius red (PR) staining (Figure 1B). Under light microscopy, a fibrillar matrix mesh was evident after 2 h of incubation (37 °C) post spraying (Figure 1B inset). Accordingly, this collagen concentration was deemed optimal for the retention of aerosolisation and gelation capacities and was used in all subsequent cell experiments. 

GFAP-DAB-peroxidase staining was technically validated by staining of control, sprayed astrocytes (minus hydrogel). Staining of coverslip (glass-adhered) sprayed astrocytes showed characteristic flattened, polygonal astrocytic morphologies (Figure 1C). This was further validated using GFAP immunofluorescence to stain sprayed astrocytes (minus hydrogel) (Figure 1D) which showed similar staining profiles. Immunocytochemical co-staining of intra-gel astrocytes using the PR-DAB-peroxidase technique showed suspended cells throughout the collagen biomatrix at 24 h and 2 weeks post spray (Figure 1E,F). Microscopic observations revealed a marked tendency for intra-gel astrocytes to organise into glial clusters (putative gliospheres) when sprayed in hydrogels (Figure 1E,F).

### 3.2. Sprayed Intra-Gel Astrocytes Show High Viability

**Figure 2:**
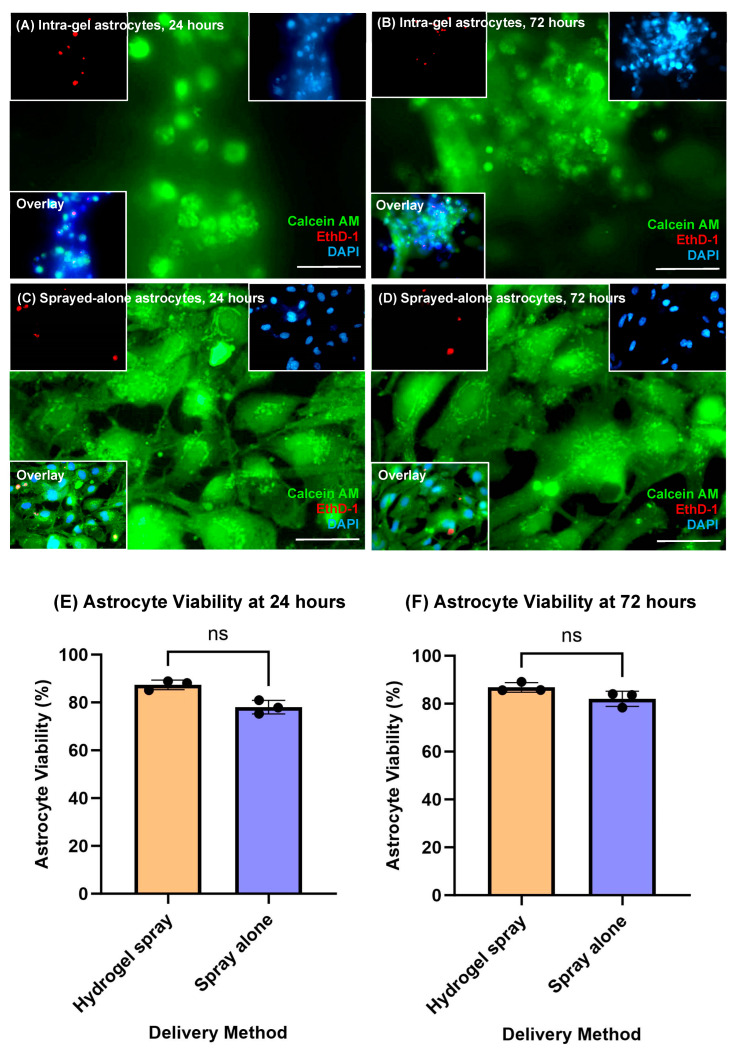
**Sprayed astrocytes remain viable within hydrogels.** Viable astrocytes are stained with Calcein AM (green), dead astrocytes with EthD-1 (red) and nuclei with DAPI (blue). (**A**,**B**) Representative, fluorescent images showing live–dead stained, hydrogel-sprayed intra-gel astrocytes at (**A**) 24 h post spray, and (**B**) 72 h post spray. (**C**,**D**) Representative, fluorescent images showing live–dead stained, sprayed-alone astrocytes at (**C**) 24 h post spray, and (**D**) 72 h post spray. (**E**,**F**) Bar charts displaying the comparative proportion of live astrocytes between hydrogel sprayed and sprayed alone at (**E**) 24 h of culture post spray, and (**F**) 72 h of culture post-spray. No significant differences in cell survival were seen at 24 h or 72 h post spray between hydrogel-sprayed and sprayed-alone astrocytes (*p* > 0.05, non-parametric Mann–Whitney tests, *n* = 3). All scale bars = 50 μm.

At 24 h, astrocyte viability was found to be 87.36 ± 1.98% and 78.04 ± 2.85% for hydrogel-sprayed and sprayed-alone cells, respectively (Figure 2A,C,E). Cell viability was not found to be statistically different between delivery methods (*p* = 0.1000, non-parametric Mann–Whitney test, *n* = 3). At 72 h, astrocyte viability remained high and was not statistically different across both delivery methods (>ca 80%), with average viabilities of 86.82 ± 1.99% versus 82.02 ± 3.16% after being sprayed in hydrogel and sprayed alone, respectively (Figure 2B,D,F; *p* = 0.1000, *n* = 3).

### 3.3. Sprayed Intra-Gel Astrocytes Proliferate, Retain Marker Expression and Tend to Cluster (Putative “Gliospheres”)

**Figure 3:**
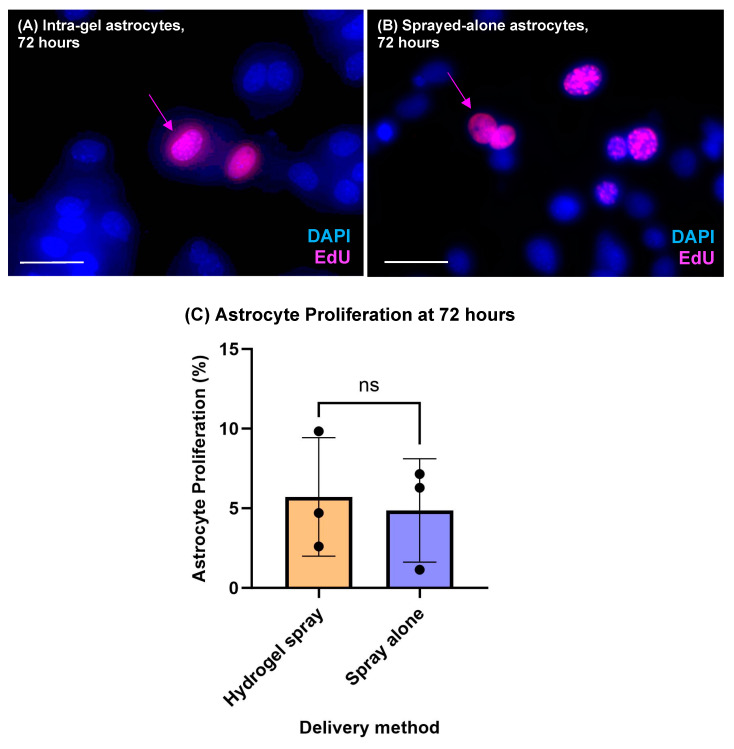
**Sprayed astrocytes demonstrate proliferation within a hydrogel biomatrix.** Proliferating astrocyte nuclei are stained with EdU (pink), and all cell nuclei with DAPI (blue). (**A**,**B**) Representative, double merged fluorescent images showing (**A**) EdU-stained hydrogel-sprayed intra-gel astrocytes cultured for 72 h post spray, and (**B**) EdU-stained sprayed-alone astrocytes cultured for 72 h post spray. Pink arrows indicate doublets of daughter cells indicative of a recent cell division. (**C**) Bar chart displaying the comparative proportion of proliferating astrocytes between hydrogel sprayed and sprayed alone with no significant differences in cell proliferation after 72 h of culture post spray (*p* > 0.05, non-parametric Mann–Whitney tests, *n* = 3). All scale bars = 50 μm.

**Figure 4:**
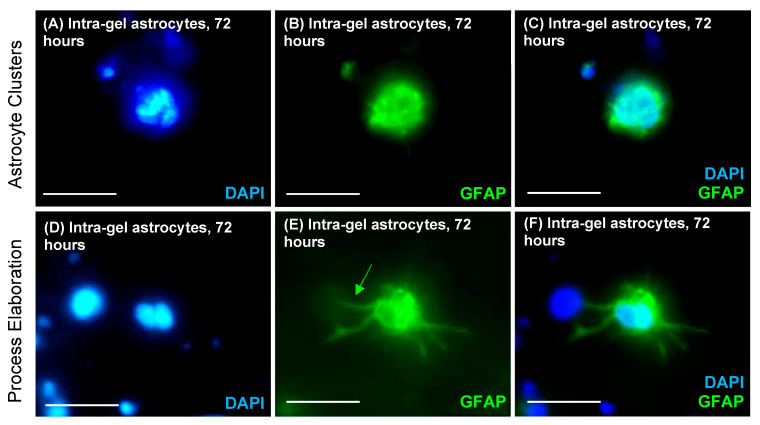
**Sprayed astrocytes have the tendency to form glial clusters (gliospheres) within hydrogels.** (**A**–**F**) Representative, fluorescent images showing sprayed intra-gel astrocytes forming glial clusters following 72 h of culture post spray. (**A**,**D**) DAPI-stained nuclei (blue) within glial cell clusters. (**B**,**E**) Intra-gel astrocytes stained for GFAP (green) forming glial cell clusters. (**C**,**F**) Double-merged images of (**A**/**B**,**D**/**E**), shown to confirm that the glial spheres in (**B**,**E**) are comprised of the multiple nuclei shown in (**A**,**D**) Astrocyte process elaboration from a glial cluster (green arrow). All scale bars = 20 μm.

Intra-gel astrocyte proliferation was evident post spray (5.71 ± 3.71%) and cell doublets of hydrogel-encapsulated EdU-stained cells were observed (inferred to represent recent cell divisions with close spatial proximity of daughter cells) (Figure 3A). Astrocytes sprayed alone had similar proliferative rates of 4.87 ± 3.24%. No significant difference in proliferation was seen between hydrogel-sprayed intra-gel astrocytes and sprayed-alone astrocytes at 72 h post spray (*p* = 0.999, *n* = 3, non-parametric Mann–Whitney test) (Figure 3C). Sprayed intra-gel astrocytes retained GFAP expression (Figure 4B,E). Morphological observations further confirmed that sprayed intra-gel astrocytes tended to form clusters (putative gliospheres) composed of multiple cells when sprayed within hydrogels (Figure 4A–C). Evidence of astrocytic process elaboration from these clusters was also observed (Figure 4D–F).

## 4. Discussion

This proof-of-concept study has demonstrated the feasibility of hydrogel encapsulation of cells for neural cell spray delivery. The method appears safe with high viability of sprayed cells and retention of indicators of physiological normalcy, such as cell marker expression and process elaboration (Figure 4). Our data show that encapsulation and delivery of cells within a hydrogel spray system do not negatively impact cell viability (Figure 2), which offers advantages for cell therapy given the protective benefits of hydrogel encapsulation. Astrocytes also retained the ability to proliferate following their delivery within a hydrogel spray with proliferation rates of 5.71 ± 3.71% versus 4.87 ± 3.24% identified for hydrogel-sprayed and sprayed-alone astrocytes, respectively (Figure 3). These proliferation rates are in line with existing studies exploring astrocytic responses to brain injury which have shown astrocytic proliferation rates of 1–2% [37,38]. A further study identified that, after 48 h in a serum-free medium, 92–95% of astrocytes are quiescent [39].

While cell viability was not increased versus spray alone, the gel-spray format could offer an augmented approach for cell therapy given the protective mechanobiological benefits of hydrogel encapsulation. Our data require further confirmation with in vivo cell delivery to confirm such advantages. In particular, in vivo comparison of cell viability with injection methods versus hydrogel sprays is required to assess the predicted mechanical and biological protection of cells. Refinement and optimisation of the hydrogel spray is also required for clinical translation. This includes establishing the most appropriate and biocompatible hydrogel material for use in spray devices. For example, natural hydrogels such as collagen possess excellent biocompatibility, mimic host extracellular matrix and limit inflammatory reactions at the time of delivery [40,41]. Conversely, synthetic hydrogels show greater compatibility with modified engineering processes to promote cell growth, differentiation, angiogenesis and induce chemotactic effects [40]. Due to the simplicity of the commercial device used, spray velocity was unable to be controlled in this study. With spray impact being dependent on both spray pattern distribution and spray angle (expressed as force/area, N/m^2^), these parameters can be refined in the future, along with the spray canisters, to reduce spray impact and shear forces on delivered transplant cells. 

Sprayed intra-gel astrocytes demonstrated a tendency to form glial clusters in hydrogel matrices (Figure 4). The reasons underpinning this clustering behaviour are unclear. Astrocytes sprayed in solution alone formed adherent monolayers, so spray droplets leading to cell clusters seems unlikely. We speculate that loosely aggregated cells during spraying attach to each other post spray and further divide to form larger, spherical glial clusters. Interestingly, such clustering/sphering of intra-gel cells may offer a further advantage of hydrogel–cell spray systems. For example, neurospheres (composed of daughter NSCs) have the advantage of improved cell protection from host inflammatory responses, with greater levels of paracrine trophic support between single NSCs [42]. Similar beneficial protection of sprayed clustered intra-gel astrocytes may also occur post transplantation. It is unclear what the fate of encapsulated, sphere-forming cells is following progressive biomatrix degradation and potential cell ‘release’ in the brain; this requires further investigation. Collectively, hydrogel-enriched sprays could offer ‘tri-layered’ cell protection that could enhance the regenerative potential of transplant neural cell populations. This includes mechanical support during the cell delivery process; formation of supportive/protective glial clusters; and biological isolation from host immune responses within the biomatrix, once in situ at the site of cell delivery [43].

Future applications of hydrogel neural cell sprays include improved delivery of neural transplant cells to locations distant from the site of cell production for clinical/experimental use. Future work could explore the use of thermosensitive hydrogels where cross-linking and gelation is induced at body temperatures post spray [44] combined with simple storage of hydrogel solutions in inexpensive refrigerators. Canton et al. explored the development of a human pluripotent stem cell (hPSC)-enriched thermo-responsive PGMA55-PHPMA135 hydrogel and observed that encapsulated PSCs entered into a quiescent G_0_ state (diapause), remaining viable for at least 14 days [45]. The ability to store intra-gel cell sprays without complex and expensive cryopreservation can aid in long-distance transport of hydrogel-encapsulated stem cells to experimental or clinical facilities. 

A potential drawback of cell sprays is the relatively superficial delivery of transplanted cells versus the delivery of cells to deeper areas by injection. Accordingly, the approach may offer greater benefits for delivery in areas such as the spinal cord versus deeper brain regions. Exogenously transplanted stem cells can also migrate to injury sites—a process termed ‘pathotropism’ where cells are chemotactically attracted to pathology foci [46]. Kelly et al. demonstrated successful migration of human foetal NSCs transplanted into ischaemic cortices of rats following middle cerebral artery occlusion, with cells migrating long distances (1.2 mm) towards the lesion [47]. Further, hydrogel matrices have been shown to promote stem cell migration (for example from the subventricular zone) which may assist in the migration of transplanted cells to deeper sites of injury [48].

We demonstrated the use of a dual immunocytological staining technique to co-visualise intra-gel cells and the hydrogel matrix. Visualisation of collagen-based materials has been challenging due to the inconsistent auto-fluorescence of collagen fibres involving fluorescence immunostaining [49]. This has the limitations of samples fading over time, the requirement for cool and dark storage and the reliance on expensive fluorescence microscopes. Simultaneous co-visualisation of both hydrogels and cells has to date involved the use of high-resolution scanning electron microscopy (HR-SEM) [50]. Despite achieving subcellular resolutions, HR-SEM is technically challenging and expensive; sample preparation requires specimen dehydration and coating in conductive materials [51]. To date, polarising light microscopy has utilised birefringence patterns to visualise PR-stained collagen sections, although this requires complex analysis to identify fibre orientation [52]. Therefore, there is a need for a technically simple protocol to co-visualise collagen biomaterials and intra-gel cells. Our method offers the advantages of simple storage and longevity of samples, alongside inexpensive light microscopy for sample visualisation. We combined simple PR-dye with immunocytological DAB-staining for an astrocyte marker (GFAP), achieving visualisation of both collagen fibres and intra-gel cells with light microscopy alone. This combinatorial technique offers a novel, technically simple and inexpensive protocol to allow co-visualisation of collagen biomaterials and intra-gel cells. It also offers applicability to the visualisation of other three-dimensional collagen constructs and intra-collagen cell behaviour and responses.

## 5. Conclusions

This in vitro feasibility study has demonstrated that the development of a hydrogel neural cell spray delivery method is feasible. To our knowledge, this cell delivery approach has not been trialled previously for neural transplantation. Such a device could offer an innovative yet inexpensive and technically simple technique to overcome limitations with current neural transplant cell delivery methods. The approach appears safe with high cell viability and parameters of physiological normalcy retained. Further studies are required to test the in vivo safety of the approach, and to optimise hydrogel and device capabilities. However, the initial report that the benefits of cell spray delivery can be fused with polymer encapsulation could offer a novel approach to augment regenerative cell therapy.

## Figures and Tables

**Table 1:** **Preparation of hydrogels and qualitative analysis of varying hydrogel concentrations for spray suitability.** Hydrogels of 0.1% (1 mg/mL), 0.3% and 0.6% were analysed for ease of spray aerosolisation, spray consistency, blockage of spray tubing and gelation capacity.

Collagen Concentration	Spray Consistency	Tubing Blockage	Gelation Capacity
**0.1% Hydrogels**	Easily sprayable, no increased resistance, consistent sprays.	No blockage of spray tubing observed.	No gelation observed at any time points.
**0.3% Hydrogels**	Sprayable with minor increased resistance. Consistent sprays.	No blockage of spray tubing observed.	Fibrillary gelation observed after 2 h of incubation at 37 °C
**0.6% Hydrogels**	Sprayable but with high levels of resistance. Inconsistent low-volume sprays.	Evidence of tubing blockage observed.	Fibrillary gelation observed after 2 h of incubation at 37 °C

## Data Availability

The data presented in this study are available on request from the corresponding author.
